# Mundgesundheitskompetenz von Menschen mit Migrationshintergrund – Erste Auswertungen der MuMi-Studie

**DOI:** 10.1007/s00103-021-03371-4

**Published:** 2021-07-01

**Authors:** Kristin Spinler, Marie-Theres Weil, Richelle Valdez, Carolin Walther, Demet Dingoyan, Udo Seedorf, Guido Heydecke, Berit Lieske, Christopher Kofahl, Ghazal Aarabi

**Affiliations:** 1 Poliklinik für Zahnärztliche Prothetik, Zentrum für Zahn‑, Mund- und Kieferheilkunde, Universitätsklinikum Hamburg-Eppendorf, Martinistraße 52, Gebäude Ost 58, 20246 Hamburg, Deutschland; 2 Institut für Medizinische Soziologie, Zentrum für Psychosoziale Medizin, Universitätsklinikum Hamburg-Eppendorf, Hamburg, Deutschland

**Keywords:** Migration, Mundgesundheit, Mundgesundheitskompetenz, Zahnmedizin, Mundgesundheitswissen, Migration, Oral health, Oral health literacy, Dentistry, Oral health knowledge

## Abstract

**Hintergrund:**

Erste Studien heben den Migrationshintergrund von Menschen in Deutschland als eigenständigen Risikofaktor für eine mangelhafte Mundgesundheit hervor. Ein wichtiger Einflussfaktor könnte hierbei eine niedrigere Mundgesundheitskompetenz von Menschen mit Migrationshintergrund sein.

**Ziel:**

In diesem Artikel werden Ergebnisse zur Mundgesundheitskompetenz und Mundgesundheit aus der MuMi-Studie (Förderung der Mundgesundheit und Mundgesundheitskompetenz von Menschen mit Migrationshintergrund) vorgestellt.

**Material und Methoden:**

In 40 Hamburger Zahnarztpraxen wurden von Patient*innen mit und ohne Migrationshintergrund Daten zu Soziodemografie, Mundgesundheit und Mundgesundheitskompetenz erhoben. Der Zusammenhang zwischen Mundgesundheitskompetenz und Mundgesundheit wurde mittels logistischer Regressionen berechnet. Potenzielle Einflussfaktoren wurden schrittweise in die Berechnungsmodelle eingefügt.

**Ergebnisse:**

Die Gruppen mit und ohne Migrationshintergrund unterschieden sich signifikant hinsichtlich ihrer Mundgesundheitskompetenz und ausgewählter klinischer Parameter ihrer Mundgesundheit (Approximalraum-Plaqueindex und Kariessanierungsgrad). Die logistischen Regressionsanalysen zeigen einen deutlichen Zusammenhang zwischen Migrationshintergrund, Mundgesundheitskompetenz und Mundhygiene auch unter Berücksichtigung von Bildung und sozioökonomischem Status.

**Diskussion:**

Der Migrationshintergrund stellt einen eigenständigen Indikator für eine niedrige Mundgesundheitskompetenz und schlechtere Mundgesundheit dar. Dieser Umstand sollte stärker in den Fokus von Forschung und politischen Entscheidungen rücken, um die mundgesundheitliche Chancengleichheit in Deutschland zu erhöhen.

**Zusatzmaterial online:**

Zusätzliche Informationen sind in der Online-Version dieses Artikels (10.1007/s00103-021-03371-4) enthalten.

## Abstract (en)

**Background:**

First investigations indicate a migration background of residents in Germany as a discrete risk factor for poor oral health. A lower level of oral health literacy among people with a migration background is considered a reason worthy of being investigated.

**Aim:**

This article presents results on oral health literacy and oral health gained from the MuMi study (promoting oral health and oral health literacy of people with a migration background).

**Methods:**

The oral health and oral health literacy as well as the sociodemographics of patients with and without migration background were examined in 40 dental surgeries in Hamburg, Germany. Associations between migrant status, oral health, and oral health literacy were analyzed with logistic regressions. Potential confounders were gradually integrated into the multivariate analyses.

**Results:**

Patients with and without a migration background differed significantly in oral health literacy and clinical parameters of oral health (approximal plaque index and degree of caries restoration). The logistic regression analysis revealed highly significant associations between migration background, oral health literacy, and oral hygiene, while also accounting for education and socioeconomic status.

**Discussion:**

Migration background constitutes a discrete risk factor for lower oral health and oral health literacy for these relevant population groups. This fact needs stronger reflection in further research and political decision-making in order to promote equality of oral health opportunities.

## Einleitung

In Deutschland hat etwa jede 4. Person (26 %) einen Migrationshintergrund [1]. Gemäß der Definition des Statistischen Bundesamts sind dies Menschen, die entweder selbst nach Deutschland immigriert sind oder mindestens ein Elternteil haben, das nicht mit deutscher Staatsbürgerschaft geboren wurde [1]. Ein Migrationshintergrund kann sowohl ein positiver als auch ein negativer Indikator für die Gesundheit eines Menschen sein [2, 3]. Im Bereich der Mundgesundheit hat sich der Migrationsstatus bisher allerdings als Risikofaktor erwiesen, wie wissenschaftliche Erhebungen an Kindern, Jugendlichen und Senior*innen ergaben [4, 5]. Dabei konnte der Migrationshintergrund, der stark mit dem sozioökonomischen Status (SES) und dem Bildungsniveau korreliert [2, 3, 6], als ein eigenständiger Risikofaktor identifiziert werden [4, 5]. Menschen mit Migrationshintergrund (im Folgenden: MmM) zeigten in diesen Untersuchungen eine signifikant schlechtere Mundgesundheit als diejenigen ohne Migrationshintergrund (im Folgenden: MoM; [4, 5, 7, 8]). Auch in anderen Ländern lässt sich dieser Zusammenhang erkennen [9–12]. Die Datenlage zur Mundgesundheit von Kindern und Jugendlichen mit Migrationshintergrund in Deutschland kann als befriedigend bewertet werden [7, 13]. Für die Erwachsenenbevölkerung mit Migrationshintergrund ist die Datenlage jedoch noch unzureichend, insbesondere im differenzierten Blick auf einzelne Herkunftsregionen [4].

Die Mundgesundheit ist stark von den psychologischen Eigenschaften einer Person abhängig, insbesondere Gesundheitswissen, Gesundheitsverhalten und Motivation [14]. Diese sind zugleich wesentliche Dimensionen der Gesundheitskompetenz, hier konkreter der *Mund*gesundheitskompetenz [15, 16]. Basierend auf der Definition der *allgemeinen* Gesundheitskompetenz umfasst diese die Fähigkeit von Individuen, grundlegende mundgesundheitsrelevante Informationen zu verstehen, zu verarbeiten und anzuwenden [17]. Eine Erhebung von Baskaradoss et al. (2018) bestätigt diesen Zusammenhang, indem sie aufzeigt, dass Personen mit niedriger Mundgesundheitskompetenz eine schlechtere Mundgesundheit (z. B. signifikant höhere Anzahl fehlender Zähne, eine niedrigere Anzahl gefüllter Zähne und eine höhere Prävalenz der schweren Form der Parodontitis) als die Vergleichsgruppe mit hoher Mundgesundheitskompetenz aufwiesen [16]. Die derzeitige Gesamtstudienlage zeigt insgesamt heterogene Ergebnisse und kann somit einen unabhängigen Einfluss der Mundgesundheitskompetenz auf die Mundgesundheit selbst nicht eindeutig beweisen [15, 18]. Mutmaßlich ist dies auf die unterschiedlichen Operationalisierungen von Mundgesundheitskompetenz (Oral Health Literacy, OHL) in den unterschiedlichen Studien zurückzuführen. Untersuchungen, in denen gezielt die Mundgesundheitskompetenz von MmM untersucht wurde, existieren bisher kaum [19, 20]. Dies mag mitunter daran liegen, dass die Gesundheitskompetenz im Allgemeinen als komplexes, multidimensionales Konstrukt nicht leicht zu messen ist [21] und die Mundgesundheitskompetenz von MmM im Besonderen bisher nicht im Fokus des politischen und wissenschaftlichen Interesses stand [22]. „Mundgesundheit und Migration“ wird mittlerweile in der gesundheitspolitischen Diskussion [23] und medizinethischen Sicht [24] in Deutschland als wichtiger Themenbereich in den Maßnahmen zur Stärkung der mundgesundheitlichen Chancengleichheit anerkannt. Zur Erreichung dieses Ziels besteht jedoch erheblicher Forschungs- und Handlungsbedarf, damit die Datenlage verbessert und mögliche Interventionen zielgerichtet angegangen werden können.

Die wenigen vorliegenden internationalen Studien weisen darauf hin, dass MmM bzw. ethnische Minderheiten bezüglich der Mundgesundheitskompetenz oder ihrer einzelnen Komponenten – wie das Wissen über Mundgesundheit oder die Befähigung, schriftliche Informationen zu erfassen – im Allgemeinen schlechter abschneiden als die jeweilige Mehrheitsbevölkerung [25, 26]. Es erscheint somit plausibel, dass eine niedrigere Mundgesundheitskompetenz ausschlaggebend für die mundgesundheitlichen Unterschiede in diesen beiden Kollektiven sein könnte. Diese Hypothese zu prüfen, ist unter anderem ein Ziel der MuMi-Studie (Förderung der Mundgesundheit und Mundgesundheitskompetenz von Menschen mit Migrationshintergrund), die seit Juli 2018 über einen Zeitraum von dreieinhalb Jahren am Universitätsklinikum Hamburg-Eppendorf (UKE) durchgeführt wird. Im Rahmen der Studie wird untersucht, ob durch ein digitales migrationssensibles Präventionsprogramm (MuMi-App), das innerhalb des Projekts entwickelt wurde, die Mundgesundheitskompetenz und folglich die Mundgesundheit von MmM verbessert werden können (www.uke.de/mumi/).

In diesem Artikel werden erste Ergebnisse zur Mundgesundheitskompetenz und Mundgesundheit von Teilnehmenden der MuMi-Studie mit und ohne Migrationshintergrund im Vergleich vorgestellt.

## Methodik

Die vorliegende Arbeit basiert auf den Daten der ersten 6 Monate (*N* = 724) der clusterrandomisierten kontrollierten MuMi-Studie. Die Rekrutierung ist zum Zeitpunkt der Erstellung dieser Publikation noch nicht abgeschlossen. Wie in vielen derzeitigen Studien beeinflusst die Coronakrise die Rekrutierung und Datenerhebung auch hier in erheblichem Maße [27] und erfordert eine Verlängerung des Erhebungszeitraums.

Die konsekutive Rekrutierung, Befragung und Untersuchung der Stichprobe, fand im Zeitraum von Dezember 2019 bis Juni 2020 in 40 niedergelassenen Zahnarztpraxen in Hamburg, überwiegend im Bezirk Mitte, statt. Mit einem Migrant*innenanteil von über 49 % ist dieser Bezirk der einwanderungsstärkste Hamburgs und somit als Modellregion sehr gut geeignet [28]. Es wurden Patient*innen eingeschlossen, die mindestens 18 Jahre alt sind, ein Handy oder Tablet nutzen und mindestens eine der Sprachen Deutsch, Englisch, Russisch, Arabisch oder Türkisch verstehen. Alle Teilnehmenden haben eine Einverständniserklärung sowie eine Datenschutzerklärung unterschrieben und einen Fragebogen zur Mundgesundheitskompetenz, zur Soziodemografie und zum Migrationshintergrund ausgefüllt. Der Fragebogen ist im Onlinematerial zu diesem Beitrag zu finden. Alle Dokumente standen in den 5 zuvor genannten Sprachen zur Verfügung. Die anschließend durchgeführte zahnmedizinische Untersuchung umfasste den Zahnstatus, den Approximalraum-Plaqueindex (API), als Messgröße zur Bewertung der Plaqueansammlung in den Zahnzwischenräumen, und den Sulkus-Blutungs-Index (SBI), der das Auftreten einer Blutung im Sulkus nach Sondierung angibt. Das Praxispersonal wurde vor Studienbeginn von Zahnärzt*innen des MuMi-Projektteams kalibriert. Die Zahnarztpraxen erhielten als Aufwandsentschädigung 50 €, die Teilnehmenden ein Mundhygieneset (Zahnbürsten, Mundspülung, Zahnseide).

### Soziodemografie und Migrationshintergrund

Alter, Geschlecht, Bildungsniveau und Äquivalenzeinkommen (Haushaltsnettoeinkommen korrigiert nach Haushaltsgröße) wurden als soziodemografische Messgrößen erhoben und für diese Publikation analysiert. Aus erhebungsökonomischen Gründen waren die genaue Haushaltszusammensetzung und das Alter der Kinder für die Berechnung des Äquivalenzeinkommens nicht verfügbar. Ein weiteres Haushaltsmitglied wurde in Ehen und Partnerschaften mit Faktor 0,5 eingerechnet, jedes weitere mit 0,4 als grob geschätzter Faktormittelwert, da Kinder unter 14 Jahren üblicherweise nur mit Faktor 0,3 eingerechnet werden [29]. Das Bildungsniveau wurde auf Basis der International Standard Classification of Education 2011 [30] bestimmt und in die 3 Niveaus niedrig, mittel und hoch unterteilt.

Der Migrationshintergrund wurde mittels Abfrage des eigenen und des Geburtslandes beider Elternteile erhoben. Für die hier vorliegenden Analysen wurden Proband*innen, die entweder selbst nach Deutschland immigriert sind oder mindestens ein Elternteil haben, das nicht in Deutschland geboren wurde [1], als MmM kategorisiert. Alle anderen Proband*innen werden als MoM aufgeführt.

### Mundgesundheitskompetenz

Die Mundgesundheitskompetenz wurde mittels eines für die MuMi-Studie entwickelten Fragebogens, dem Oral Health Literacy Profile (OHLP), erfasst. Das OHLP basiert auf dem aktuellen wissenschaftlichen Kenntnisstand zur Mundgesundheit und den evidenzbasierten Empfehlungen zur Mundhygiene [31–34]. Es umfasst die Module: Mundgesundheitsverhalten (8 Items: z. B. allgemeine Fragen zur praktizierten Mundhygiene der Befragten, wie Zahnputzdauer und Inanspruchnahme zahnärztlicher Leistungen), Wissen über Mundgesundheit (10 Items: z. B. zahnfreundliche Ernährung, Zusammenhang zwischen Mundgesundheit und allgemeiner Gesundheit), Wissen über zahnmedizinische Versorgung in Deutschland (5 Items: z. B. welche zahnmedizinischen Leistungen von Krankenkassen übernommen werden) und die emotionale Komponente der Mundgesundheit (2 Items: Angst vor hohen Kosten oder vor Schmerzen als Barrieren für Zahnarztbesuche). Zusätzlich beinhaltet das OHLP 3 Einzelfragen zum Grund des letzten Zahnarztbesuches und zu Selbsteinschätzungen der eigenen Mundgesundheit sowie des eigenen Wissens über die Mundgesundheit, die nicht in die Berechnung der Gesamtskala eingehen. Der Fragebogen wurde, in Anlehnung an den Ansatz TRAPD (Translation, Review, Adjudication, Pretesting, Documentation) nach Harkness (2008), in einem mehrstufigen Verfahren mit professionellen Übersetzer*innen vom Deutschen in die Sprachen Englisch, Russisch, Arabisch und Türkisch übersetzt [35].

Die Items aus den Verhaltens- und Wissensmodulen sowie der emotionalen Komponente zählten je einen Punkt für eine richtige Antwort. Der Prozentwert der erreichten Punkte relativ zur Gesamtzahl der jeweiligen Modulitems wurde in eine Skala von 0 bis 100 umgewandelt, wobei höhere Werte eine bessere Mundgesundheitskompetenz widerspiegeln.

### Zahnmedizinische Untersuchung

Zur Bewertung der Mundgesundheit wurden folgende klinischen Parameter durch die niedergelassenen Zahnärzt*innen gemessen: 1. der Zahnstatus, 2. der API und 3. der SBI. In der Methodik- und Ergebnisdarstellung wird auf Zahnstatus und API eingegangen. Der API wird als Indikator für die Mundhygiene herangezogen [36]. Er misst das Vorhandensein von Plaque in den Zahnzwischenräumen. Plaque ist ein Zahnbelag, der aus Speichel, bakteriellen Stoffwechselprodukten, Speiseresten und Bakterien besteht. Er ist grundsätzlich sehr gut durch sorgfältige Zahnpflege vermeidbar und somit ein guter Indikator für das individuelle Mundpflegeverhalten. Plaque stellt einen entscheidenden Faktor bei der Entstehung von Erkrankungen wie Karies und Parodontitis dar [37]. Der API wurde ohne Anfärben der Zahnzwischenräume visuell beurteilt (Plaque vorhanden: Ja/Nein). Die Berechnung des API erfolgt über die Formel: $\frac{\text{Summe der positiven Plaquemessungen }\mathrm{x}100}{\text{Gesamtzahl vorhandener Approximalraum}-\text{Messpunkte}}$. Hieraus ergibt sich der API (Werte von 0 bis 100), wobei niedrigere Werte eine bessere Mundhygiene anzeigen. Aus den Indexwerten werden 4 Kategorien gebildet: optimal < 25 %, gut 25–39 %, mäßig 40–69 %, unzureichend 70–100 % [38].

Als Indikator für den Kariesbehandlungsbedarf wurde der Kariessanierungsgrad herangezogen, der aus den erhobenen Daten des Zahnstatus wie folgt berechnet wird: $(\frac{\text{Anzahl gef"ullter Z"ahne}}{\text{Anzahl kari"oser}+\text{gef"ullter Z"ahne}}\times$ 100) [39]. Je höher der Anteil an bereits sanierten Zähnen im Verhältnis zu den kariösen, noch zu füllenden Zähnen ist, desto höher ist der Sanierungsgrad.

### Statistische Analysen

Zur Berechnung von Gruppenunterschieden zwischen MmM und MoM hinsichtlich Demografie, Bildung, Äquivalenzeinkommen, Mundgesundheitskompetenz, klinischer Parameter der Mundgesundheit (API, Kariessanierungsgrad), Inanspruchnahme zahnärztlicher Dienstleistungen und Zahnputzdauer wurden Χ^2^-Tests (bzw. der exakte Fisher-Test) und Mann-Whitney-U-Tests genutzt. Für die Zusammenhänge zwischen Mundgesundheitskompetenz und API sowie der Selbsteinschätzung des Wissens über Mundgesundheit wurde der Spearman-Korrelationskoeffizient für ordinalskalierte Daten verwendet.

Der Zusammenhang zwischen Mundgesundheitskompetenz und API wurde mittels logistischer Regressionen berechnet. Hierfür wurden der OHLP-Gesamtindex (überdurchschnittlicher OHLP-Index: Ja/Nein) und die API-Skala (optimale oder gute Mundhygiene: Ja/Nein) dichotomisiert. Potenzielle Störfaktoren wurden schrittweise in die Berechnungsmodelle eingefügt. Aufgrund einer hohen Nichtantwortrate bei der Frage zum Haushaltsnettoeinkommen (38 %) wurden die multivariaten Analysen unter Einschluss des Äquivalenzeinkommens wegen der hierdurch deutlich reduzierten Stichprobengröße gesondert durchgeführt.

Alle Berechnungen erfolgten mit SPSS™ Versionen 26 und 27. Die Irrtumswahrscheinlichkeit (bzw. statistische Signifikanz) wurde für alle Verfahren auf 5 % festgelegt.

## Ergebnisse

### Stichprobenbeschreibung

Die hier präsentierten Ergebnisse beziehen sich auf Analysen der ersten 724 Proband*innen der MuMi-Studie (Tab. 1). Die Proband*innen haben ein Durchschnittsalter von 39,5 Jahren (SD = 13,5) und sind mehrheitlich Frauen (58,8 %). Das Bildungsniveau ist relativ gleichmäßig auf alle 3 Kategorien (niedrig, mittel, hoch) verteilt, wobei die größte Gruppe (39,9 %) dem eher niedrigen Bildungsniveau zuzuordnen ist. Das durchschnittliche Äquivalenzeinkommen beträgt 1808 € (SD = 1022 €). Mit 38,1 % (*n* = 161) bewegen sich die meisten Proband*innen in der mittleren Kategorie von 1000 € bis < 2000 €. 22,4 % (*n* = 162) der Befragten machten keine Angabe zum Haushaltsnettoeinkommen, hierbei deutlich mehr MmM (25 %) als MoM (14 %). Die MuMi-Sampling-Strategie sieht einen Migrant*innenanteil von 80 % vor. In dieser ersten Auswertungsstichprobe erfüllen 563 Proband*innen (77,8 %) dieses Kriterium. Von diesen sind 429 Personen (59,3 %) selbst nach Deutschland immigriert und 134 (18,5 %) haben mindestens ein Elternteil, das nicht in Deutschland geboren wurde. Die Aufenthaltsdauer in Deutschland lag zum Zeitpunkt der Erhebung bei 1–51 Jahren, im Durchschnitt bei 15,5 Jahren (SD = 12,58). Die am stärksten vertretenen Herkunftsregionen sind Osteuropa innerhalb der Europäischen Union (EU; 36,8 %), der Mittlere Osten (28,6 %), Osteuropa außerhalb der EU (16,9 %), gefolgt von Westeuropa (7,4 %) und kleineren Gruppen aus Ozeanien, Amerika und Afrika.

**Table 1:** 

	Gesamt	MoM	MmM	
	*N* = 724	*N* = 161	*N* = 563	
	Durchschnitt (±SD) oder *N* (%)	Durchschnitt (±SD) oder *N* (%)		p
**Demografie**	–	–	–	–
Alter (Jahre)	39,5 (±13,5)	44,0 (±14,1)	38,7 (±13,4)	**<** **0,001**^a^
Geschlecht (weiblich)	426 (58,8 %)	98 (60,9 %)	328 (58,9 %)	0,72^b^
**Bildung (basierend auf Abschlüssen)**	–	–	–	–
*Niedriges Bildungsniveau*	277 (39,9 %)	56 (35,2 %)	221 (41,3 %)	0,39^b^
*Mittleres Bildungsniveau*	194 (28,0 %)	48 (30,2 %)	146 (27,3 %)	
*Hohes Bildungsniveau*	223 (32,1 %)	55 (34,6 %)	168 (31,4 %)	
**Äquivalenzeinkommen**^**c**^** in €**	1808 (±1022)	2277 (±1018)	1647 (±974)	***<*** ***0,001***^*a*^
*<* *1000* *€*	112 (26,5 %)	17 (15,2 %)	121 (34,3 %)	***<*** ***0,001***^*a*^
*1000– <* *2000* *€*	161 (38,1 %)	31 (27,7 %)	143 (40,5 %)	
*Mind. 2000* *€*	150 (35,4 %)	64 (57,1 %)	89 (25,2 %)	
**Selbsteinschätzung**	–	–	–	–
**… des Wissens über Mundgesundheit**^**e**^	2,82 (±0,88)	2,94 (±0,84)	2,78 (±0,89)	0,41^a^
**… der Mundgesundheit**^**e**^	3,04 (±0,86)	3,10 (±0,84)	3,03 (±0,87)	**0,05**^a^
**Fragebogen zur Mundgesundheitskompetenz (OHLP)**	52,6 (±19,2)	64,5 (±14,7)	49,2 (±19,0)	**<** **0,001**^a^
*Mundgesundheitsverhalten (8 Items)*	50,6 (±28,8)	65,2 (±24,1)	46,4 (±28,7)	**<** **0,001**^a^
*Wissen über Mundgesundheit (10 Items)*	49,0 (±30,0)	62,3 (±28,3)	45,2 (±29,5)	**<** **0,001**^a^
*Wissen über das System der zahnmedizinischen Versorgung (5 Items)*	52,5 (±28,3)	67,4 (±21,1)	48,2 (±28,7)	**<** **0,001**^a^
*Emotionale Komponente (2 Items)*	58,2 (±26,0)	63,0 (±24,3)	56,8 (±26,3)	**0,007**^a^
**Mundgesundheit – Klinische Parameter**	–	–	–	–
**API-Auswertung**	50,1 (±30,7)	38,3 (±28,4)	52,5 (±30,6)	**<** **0,001**^a^
*Optimal (<* *25* *%)*	174 (24,2 %)	60 (37,5 %)	114 (20,4 %)	**<** **0,001**^a^
*Gut (25–39* *%)*	102 (14,2 %)	29 (18,1 %)	73 (13,0 %)	
*Mäßig (40–69* *%)*	234 (32,5 %)	45 (28,1 %)	189 (33,8 %)	
*Unzureichend (70–100* *%)*	210 (29,2 %)	26 (16,3 %)	184 (32,9 %)	
**Kariessanierungsgrad**	84,3 (±28,7)	93,8 (±18,4)	81,7 (±30,6)	**<** **0,001**^a^
**Inanspruchnahmeverhalten Zahnarztbesuch innerhalb der letzten 12 Monate (Ja)**	625 (86,3 %)	148 (91,9 %)	477 (84,7 %)	**0,02**^b^
**Grund für den letzten Zahnarztbesuch**^**d**^	–	–	–	–
*Vorsorge/Kontrolle*	433 (59,8 %)	116 (72,0 %)	317 (56,3 %)	**<** **0,001**^b^
*Schmerzen/Beschwerden*	179 (24,7 %)	22 (13,7 %)	157 (27,9 %)	**<** **0,001**^b^
*Geplante Therapie*	133 (18,4 %)	28 (17,4 %)	105 (18,7 %)	0,82^b^
**Mundhygieneverhalten**	–	–	–	–
**Zahnputzdauer**	–	–	–	–
*<* *2* *min*	149 (20,6 %)	18 (11,2 %)	131 (23,3 %)	**<** **0,001**^a^
*≥* *2* *min*	575 (79,4 %)	143 (88,8 %)	432 (76,7 %)	

*API* Approximalraum-Plaqueindex, *MmM* Menschen mit Migrationshintergrund, *MoM* Menschen ohne Migrationshintergrund, *N* absolute Anzahl, *SD* Standardabweichung

^a^Mann-Whitney

^b^Chi-Quadrat

^c^*N* = 423

^d^Mehrfachantworten möglich

^e^1 schlecht bis 5 ausgezeichnet

Die Untersuchungspersonen schätzten ihren eigenen *allgemeinen* Gesundheitszustand auf einer Fünferskala von schlecht (1) bis ausgezeichnet (5) mit 3,97 (SD = 0,79) im Durchschnitt als „sehr gut“ ein. Die eigene *Mund*gesundheit wird mit einem Durchschnitt von 3,04 (SD = 0,86) auf der gleichen Antwortskala als „gut“ bewertet. Das Wissen über Mundgesundheit wird mit einem Durchschnittswert von 2,82 (SD = 0,88) ebenfalls als „gut“ eingeschätzt.

Die Mundgesundheitskompetenz der Stichprobe lag in den Messergebnissen des OHLP bei durchschnittlich 52,6 (SD = 19,18) von 100 möglichen Punkten. Die Unterdimension mit der höchsten Durchschnittspunktzahl (58,2 Punkte) ist die emotionale Komponente, die mit der niedrigsten das Wissen über Mundgesundheit (49,0 Punkte). Die Selbsteinschätzung des Mundgesundheitswissens der Proband*innen korreliert hoch positiv mit der durch das OHLP erhobenen Mundgesundheitskompetenz (rho = 0,43; *p* < 0,001).

Im Ergebnis der zahnärztlichen Untersuchungen weist die Mehrzahl der Untersuchten (61,7 %) allerdings nur eine mäßige bis unzureichende Mundhygiene auf (API). Der durchschnittliche Kariessanierungsgrad beträgt 84,3 % (SD = 28,7 %). Bezüglich des Mundpflegeverhaltens geben 67 % an, ihre Zähne mindestens zweimal am Tag zu putzen. 79,4 % nennen eine Zahnputzdauer von mindestens 2 min. 86,3 % hatten innerhalb der letzten 12 Monate eine Zahnarztpraxis aufgesucht, wobei „Vorsorge und Kontrolle“ als häufigster Grund (59,8 %) angegeben wurde.

### Gruppenunterschiede

Es zeigen sich signifikante Unterschiede zwischen den Gruppen der MmM und der MoM hinsichtlich Alter, Äquivalenzeinkommen, Mundgesundheitskompetenz, klinischer Parameter der Mundgesundheit, Selbsteinschätzung der Mundgesundheit, Zahnputzdauer und Inanspruchnahme zahnärztlicher Leistungen (Tab. 1).

In dieser Stichprobe sind die MmM (Ø = 38,7 Jahre) durchschnittlich 5,3 Jahre jünger als diejenigen ohne Migrationshintergrund (Ø = 44,0 Jahre). Sie haben außerdem mit 1647 € ein signifikant niedrigeres Durchschnittsäquivalenzeinkommen (MoM = 2277 €).

Die Gruppe der MmM schnitt im Bereich der Mundgesundheitskompetenz signifikant schlechter ab als die Gruppe der MoM: 49,2 (SD = 19,0) vs. 64,5 (SD = 14,7) Punkte im OHLP-25. Auch in allen einzelnen Unterkategorien (jeweils in einer Skala von 0–100) zeigten sich diese signifikanten Unterschiede. Die größten Differenzen zwischen MoM und MmM bestehen in den Dimensionen „Mundgesundheitsverhalten“ (Ø = 18,8 Punkte) und „Wissen über das System der zahnmedizinischen Versorgung“ (Ø = 19,2 Punkte).

3 Einzelitems der Unterkategorie Mundhygieneverhalten möchten wir hier gesondert darstellen, weil sie für die Mundgesundheit besonders bedeutsam sind. Sie zeigen, dass signifikant weniger MmM in den letzten 12 Monaten einen Zahnarzt aufgesucht hatten als MoM (84,7 % vs. 91,9 %), dabei war der Grund für den letzten Zahnarztbesuch von MoM mit 72,0 % signifikant häufiger „Vorsorge/Kontrolle“ als bei MmM (56,3 %). Weiter gaben nur 76,7 % der MmM gegenüber 88,8 % der MoM eine Zahnputzdauer von mindestens 2 min an.

Auch in den klinischen Mundgesundheitsparametern sind deutliche Gruppenunterschiede erkennbar. Mit einem API von 52,5 (SD = 30,6) und einem Kariessanierungsgrad von 81,7 % (SD = 30,6 %) zeigt die Gruppe der MmM deutlich schlechtere Werte als die der MoM, deren API bei 38,3 (SD = 28,4) und Sanierungsgrad bei 93,8 % (SD = 18,4 %) liegt. Letztere gaben als Grund für den Zahnarztbesuch häufiger „Vorsorge/Kontrolle“ und seltener „Schmerzen/Beschwerden“ (13,7 % vs. 27,9 %) an.

### Zusammenhang zwischen Mundgesundheitskompetenz und Mundhygiene

Die Korrelation zwischen API (Mundhygiene) und OHLP-25 (Mundgesundheitskompetenz) zeigt eine signifikante Assoziation zwischen höherer Mundgesundheitskompetenz und besseren Mundhygienewerten, d. h. niedrigen Werten im API (rho = −0,40; *p* < 0,001). Die logistische Regressionsanalyse bestätigt, dass eine überdurchschnittliche Mundgesundheitskompetenz (> 52,6 Punkte im OHLP) die Wahrscheinlichkeit für eine gute bis optimale Mundhygiene erhöht, auch nach der Kontrolle von Einflussfaktoren wie Migrationshintergrund, Alter, Geschlecht und Bildung (OR = 2,75; *p* < 0,001; Tab. 2). Kein Migrationshintergrund (OR = 1,75), das weibliche Geschlecht (OR = 1,71) und/oder ein hohes Bildungsniveau (OR = 1,58) erhöhen ebenfalls die Wahrscheinlichkeit einer guten bis optimalen Mundhygiene.

**Table 2:** 

	OR	KI 95 %	*p*
**Modell 1 (*n*** **=** **720)**			
Überdurchschnittlicher OHLP	3,29	2,41–4,53	**<** **0,001**
**Modell 2 (*n*** **=** **720)**			
Überdurchschnittlicher OHLP	2,88	2,07–4,00	**<** **0,001**
Kein Migrationshintergrund	1,77	1,21–2,59	**0,003**
**Modell 3 (*n*** **=** **678)**			
Überdurchschnittlicher OHLP	2,75	1,95–3,89	**<** **0,001**
Kein Migrationshintergrund	1,75	1,18–2,60	**0,006**
Alter	0,99	0,98–1,00	0,097
Geschlecht (weiblich)	1,71	1,22–2,41	**0,002**
Bildung	–	–	**–**
*Niedriges Bildungsniveau (Referenz)*	–	–	**–**
*Mittleres Bildungsniveau*	1,15	0,76–1,72	0,513
*Hohes Bildungsniveau*	1,58	1,08–2,33	**0,020**

*API* 0 unzureichende bis mäßige Mundhygiene, 1 gute bis optimale Mundhygiene, *KI* Konfidenzintervall, *OR* Odds Ratio, *OHLP* Oral Health Literacy Profile, *p* Signifikanzwert

Die logistische Regression zur Beurteilung des Zusammenhangs zwischen Äquivalenzeinkommen und Mundhygiene zeigt einen leicht signifikanten (*p* = 0,041) Zusammenhang zwischen API und überdurchschnittlichem Einkommen (> 1500 € im Monat) mit einem OR von 1,51 (KI 95 % = 1,02−2,23), der bei Hinzunahme aller anderen oben eingeführten Indikatoren auf ein OR von 1,20 (nicht signifikant) absinkt. Mit Ausnahme des OHLP (OR = 2,59; KI 95 % = 1,64−4,07; *p* < 0,001) sind allerdings auch alle anderen Indikatoren nicht signifikant, was zum Teil der deutlich reduzierten Stichprobengröße geschuldet ist.

## Diskussion

Die ersten Ergebnisse aus dem MuMi-Projekt liefern Antworten auf die Frage nach der Mundgesundheitskompetenz von MmM. Es zeigen sich deutliche Unterschiede zwischen Menschen mit und ohne Migrationshintergrund in der Mundgesundheitskompetenz, die bei MmM im Durchschnitt signifikant niedriger ist (OHLP-25 = 49,2) als bei MoM (OHLP-25 = 64,5), als auch in der Mundhygiene, die bei MmM im Durchschnitt signifikant schlechter ausfällt (API = 52,5) als bei MoM (API = 38,3). Der Sanierungsgrad ist bei MoM ebenfalls signifikant höher als bei MmM (93,8 vs. 81,7). Wie bereits in anderen Untersuchungen beschrieben [4, 5], scheint der Migrationshintergrund einen eigenständigen Einflussfaktor darzustellen, da die Unterschiede auch nach der statistischen Kontrolle für sozioökonomische und Bildungsfaktoren bestehen bleiben, wobei wir bezüglich der sozioökonomischen Faktoren einschränkend anmerken müssen, dass viele Proband*innen keine Angaben zu ihrem Einkommen machten.

Das OHLP-25 ist ein im Rahmen dieser Studie neu entwickeltes Instrument zur Einschätzung der Mundgesundheitskompetenz, das im Einklang mit dem aktuellen evidenzbasierten Wissen über Mundgesundheit und den Empfehlungen zur Mundpflege steht [31–34]. Darüber hinaus korrespondiert es mit den für Patient*innen relevanten Empfehlungen und Bedingungen der zahnärztlichen Versorgung in Deutschland. In diesem Anwendungsfall zeigt das OHLP-25 sowohl als Gesamtindex wie auch in seinen Subdimensionen sehr gute Verteilungen, die annähernd Normalverteilungen entsprechen und somit die Voraussetzung erfüllen, zwischen verschiedenen Patient*innen gut differenzieren zu können.

Der deutliche Zusammenhang zwischen Mundgesundheitskompetenz und der Mundgesundheit als solcher erscheint inhaltlich plausibel, ist aber nicht selbstverständlich. Die Untersuchungsergebnisse können als datengestützter Hinweis interpretiert werden, dass sich eine höhere Mundgesundheitskompetenz tatsächlich in einer besseren Mundgesundheit niederschlagen könnte. Das querschnittliche Design lässt es jedoch nicht zu, eine solche kausale Beziehung zu unterstellen und dieses komplexe Konstrukt der Wirkungsrichtung sowie den Einfluss weiterer Faktoren in der Tiefe zu durchleuchten. Inhaltlich scheint es allerdings nicht schlüssig, von der umgekehrten Kausalität auszugehen, dass eine schlechte bzw. gute Mundgesundheit zu einer niedrigen bzw. hohen Mundgesundheitskompetenz führen würde. Im Gegenteil könnte sogar davon ausgegangen werden, dass Patient*innen mit höherem Behandlungsbedarf aufgrund schlechter Mundgesundheit im Rahmen ihrer Behandlung ein Erfahrungswissen sammeln, das zumindest in den wissensbezogenen Dimensionen des OHLP-25 zu höheren Werten führen würde, z. B. bei Fragen zu den Behandlungsschritten bei entzündetem Zahnnerv (ein Item aus dem OHLP). Sollte dies zutreffen, wäre der ermittelte Zusammenhang zwischen OHLP und den beiden in dieser Studie verwendeten Mundgesundheitsindikatoren API und Kariessanierungsgrad in dieser Logik sogar noch unterschätzt.

**Figure 1:**
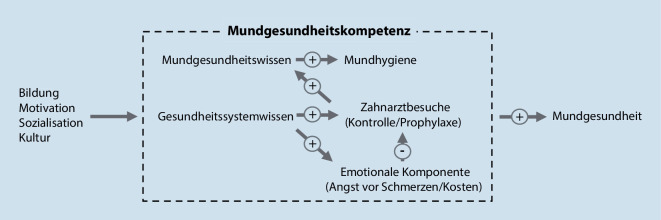

Die Ergebnisse stützen somit die These, dass sich Mundgesundheitskompetenz auf die klinische Mundgesundheit auswirkt [16, 40], wobei die einzelnen Dimensionen der Gesundheitskompetenz bereits selbst in kausal verketteten Beziehungen stehen. Das OHLP deckt als Kurzassessment nicht alle Facetten von Mundgesundheitskompetenz ab, sondern fokussiert auf Wissen und Verhalten, ergänzt um eine negativ attribuierte Motivation (Angst vor Schmerzen und Kosten) als Barriere für Zahnarztbesuche. Die beschriebenen Wechselwirkungen zwischen den Unterdimensionen der Mundgesundheitskompetenz (gemessen durch das OHLP) und Mundgesundheit sind in Abb. 1 in schematischer Form dargestellt.

Die Zahnmedizin und die allgemeine Gesundheitsbildung in Deutschland haben hinsichtlich der Entwicklung der Mundgesundheit in den letzten beiden Jahrzehnten eine wahre Erfolgsgeschichte zu verbuchen. In kaum einem anderen Gesundheitsbereich sind die Inzidenzen und Prävalenzen derart rückläufig [39]. Diese positiven Entwicklungen betreffen alle sozioökonomischen Statusgruppen. Allerdings bleibt die soziale Ungleichheit bestehen, der soziale Gradient verschiebt sich lediglich auf ein höheres Niveau [38].

Die Deutschen Mundgesundheitsstudien (DMS) konnten bislang keine Aussagen zur Mundgesundheit von MmM treffen, da der Migrationshintergrund bisher nicht erhoben wurde [38], was allerdings in der kommenden DMS VI der Fall sein wird [41]. Die ersten vorliegenden Ergebnisse aus dem MuMi-Projekt weisen die MmM als eine höchst vulnerable Zielgruppe aus, nicht nur weil sie überdurchschnittlich häufig einer niedrigeren Sozialstatusgruppe angehören, sondern auch weil sie aufgrund ihrer unterschiedlichen Sozialisation, Sprache und Bildung [42] geringere Chancen hatten und haben als die in Deutschland sozialisierte Mehrheitsbevölkerung, die Maßnahmen zur Mundgesundheitsprävention und ggf. Behandlung zu erkennen, zu verstehen und in Anspruch zu nehmen [43]. Somit sind die Migrationshintergründe zusätzliche erschwerende Faktoren hinsichtlich der Erhaltung der Mundgesundheit [4]. Demzufolge ist in dieser Konstellation unverkennbar, dass (Mund‑)Gesundheitskompetenz in erheblichem Maße auch von den gesellschaftlichen Rahmenbedingungen abhängt, insbesondere von den Anschlussmöglichkeiten, die sich dem Einzelnen bieten [44].

Offensichtlich benötigen verschiedene Bevölkerungsgruppen mit Migrationshintergrund eine eigene und zielgerichtete Ansprache [43]. In dem MuMi-Projekt versuchen wir dies mit einer Mundgesundheitspräventions-App in verschiedenen Sprachen. Diese ist natürlich nur ein Baustein von mehreren im Kontext der interkulturellen Öffnung von Zahnarztpraxen und weiterer Versorgungsinstitutionen.

### Limitationen

Es ist von einer sehr großen Heterogenität der Menschen mit Migrationshintergrund auszugehen, die es in den weiteren Analysen und künftigen Studien (z. B. durch Subgruppenanalysen) stärker zu berücksichtigen gilt. Denn die Beschreibung dieser Gruppe als ein Kollektiv im Rahmen der ersten vorgenommenen Auswertungen wird der Diversität der einzelnen Kulturkreise nicht angemessen gerecht.

Da die Rekrutierung in niedergelassenen Zahnarztpraxen erfolgte, kann eine Selektionsverzerrung nicht ausgeschlossen werden. Es konnten demnach nur Personen in die Studie eingeschlossen werden, die selbstständig eine Zahnarztpraxis aufgesucht haben. Diejenigen, die gar keine zahnärztliche Versorgung in Anspruch nehmen, können deshalb nicht repräsentiert werden. Durch das Ausfüllen der Fragebögen in der Zahnarztpraxis könnte eine „soziale Erwünschtheit“ Einfluss auf die Antworten genommen haben, z. B. bei den Fragen zum Mundhygieneverhalten. Es ist zusätzlich anzumerken, dass einige Fragen zum Mundhygieneverhalten, wie die Zahnputzdauer auf Selbstauskünften basieren, wodurch das tatsächliche Verhalten über- oder unterschätzt werden könnte.

Die Fragebögen wurden in 5 Sprachversionen angeboten. Es gab jedoch einige MmM, die keine dieser Sprachen als Muttersprache hatten. In der Regel nutzten diese die deutsche und einige die englische Version. Es ist zu vermuten, dass diesen das Verständnis der Fragen schwerer gefallen sein dürfte als denjenigen, die die Fragebögen muttersprachlich beantwortet haben. Dies könnte die Testergebnisse des OHLP negativ beeinflusst haben.

Das OHLP deckt die aus unserer Sicht wesentlichen, jedoch nicht alle Dimensionen der Mundgesundheitskompetenz ab. So sind beispielsweise die funktionelle Literalität, motivationale Aspekte und die Interaktionskompetenz keine Bestandteile des OHLP.

## Ausblick und Fazit

Die in unserer Studie eingeschlossenen MmM stellen eine äußerst heterogene Gruppe dar. Sie stammen aus vielfältigen Kulturkreisen und Gesellschaftssystemen. Die Fallzahlen sind aktuell noch nicht groß genug, um zu analysieren, inwieweit möglicherweise die spezifische Herkunft der MmM eine Rolle spielt und ob sich MmM, die in Deutschland geboren sind, hinsichtlich ihrer Mundgesundheitskompetenz und Mundgesundheit von denen unterscheiden, die aus dem Ausland zugewandert sind. Die Fragestellung ist von weitreichender Bedeutung, da kulturelle Einflüsse und die Sozialisation von eingewanderten Eltern Auswirkungen auf die Mundgesundheitskompetenz der nachfolgenden Generationen haben können. Dies könnte sich, als einer von vielen Faktoren, auch künftig auf die Unterschiede der Mundgesundheit von MoM und MmM negativ auswirken.

Mundgesundheit und Mundgesundheitskompetenz von MmM unterscheiden sich deutlich von denen der MoM. Diese Aspekte sollten dringend in den Fokus weiterer Studien rücken, um gezielte Förderungsmaßnahmen entwickeln und dadurch die mundgesundheitliche Chancengleichheit in Deutschland stärken zu können.

## Supplementary Information


