# Supplementary material for: Oral health literacy of persons with migration background—first results of the MuMi study
Source: Bundesgesundheitsblatt Gesundheitsforschung Gesundheitsschutz. 2021 Jul 1;64(8):977–85. [Article in German] doi: 10.1007/s00103-021-03371-4 (PMC8316185; doi:10.1007/s00103-021-03371-4)
Supplement: Supplementary file 1 [file 103_2021_3371_MOESM1_ESM.pdf]

Datum	Pseudonym

Fragebogen zur Mundgesundheit

Sehr geehrte Teilnehmerin, sehr geehrter Teilnehmer,

die Fähigkeit, gesundheitliche Informationen zu finden, diese zu verstehen und im Sinne der Gesundheitserhaltung anzuwenden, wird zunehmend bedeutsamer. Gesundheitskompetenz hat viele Facetten und Dimensionen. Der vorliegende Fragebogen soll uns helfen zu verstehen, was die Allgemeinbevölkerung über die Mundgesundheit und das Gesundheitssystem in Deutschland sowie über ihr Mundgesundheitsverhalten weiß. Damit kann zukünftig besser eingeschätzt werden, welche Menschen Informations- und ggf. Schulungs- und Beratungsbedarfe haben.

Die folgenden Fragen sind Multiple-Choice-Fragen. Bitte kreuzen Sie die richtige Antwort aus der jeweiligen Liste an.

Wenn Sie die Antwort auf eine Frage nicht wissen, erraten oder überspringen Sie die Frage bitte nicht, sondern markieren Sie bitte „Weiß nicht" und fahren dann mit der nächsten Frage fort. Einige Fragen sind bewusst schwierig gehalten, und nicht jeder wird die Antwort wissen, andere Fragen sind scheinbar sehr leicht.

Zusätzlich benötigen wir für unsere Auswertung noch ein paar Daten zu Ihrer Person. Wir erfragen keine persönlichen Daten wie Namen und Adressen oder den genauen Geburtstag.

Bitte verwenden Sie keine Hilfsmittel oder fragen andere Personen, sondern beantworten Sie die Fragen spontan, ohne allzu lange zu überlegen.

Viel Spaß beim Selbst-Test!

MuMi „Förderung der Mundgesundheitskompetenz und Mundgesundheit von Menschen mit Migrationshintergrund" ist ein Kooperationsprojekt der Poliklinik für Zahnärztliche Prothetik (ZMK) und des Instituts für Medizinische Soziologie (IMS) am Universitätsklinikum Hamburg-Eppendorf (UKE).

Das Projekt wird gefördert durch die gesetzlichen Krankenkassen in Rahmen des Innovationsfonds des Gemeinsamen Bundesausschusses.

Website: www.uke.de/mumi

Projektleitung:

Dr. Ghazal Aarabi, M.Sc. (ZMK)
Dr. Christopher Kofahl, Dipl.-Psych. (IMS)

Ansprechpartnerin: Kristin Spinler, M.Sc.

Addresse: UKE, Martinistraße 52, Gebäude Ost 58, 20246 Hamburg
Telefon: (040) 7410 - 54660 | Montag - Donnerstag |

Email: mumi@uke.de

Mundgesundheit & Hygiene

1 **Alles in allem, wie würden Sie Ihre allgemeine Mundgesundheit bewerten?**

○ Ausgezeichnet ○ Sehr gut ○ Gut ○ Befriedigend ○ Schlecht

2 **Wann waren Sie das letzte Mal bei einem Zahnarzt?**

- ○ Ich war noch nie zuvor bei einem Zahnarzt
- ○ Vor über einem Jahr
- ○ Innerhalb der letzten 12 Monate
- ○ Innerhalb der letzten 6 Monate
- ○ Weiß nicht

3 **Wie stark treffen die folgenden Aussagen auf Sie zu?**

**„Wenn ich zum Zahnarzt gehe, habe ich…“**	Trifft überhaupt nicht zu	Trifft nicht zu	Trifft ein wenig zu	Trifft zu	Trifft voll zu
… Angst vor hohen Kosten	○	○	○	○	○
… Angst vor Schmerzen	○	○	○	○	○

4 **Was war der Grund für Ihren letzten Zahnarztbesuch?**

- ○ Vorsorge/Kontrolle
- ○ Schmerzen/Beschwerden
- ○ Geplante Therapie
- ○ Weiß nicht

5 **Wann waren Sie das letzte Mal bei einer professionellen Zahnreinigung?**

- ○ Ich habe mir die Zähne noch nie professionell reinigen lassen
- ○ Vor über einem Jahr
- ○ Innerhalb der letzten 12 Monate
- ○ Innerhalb der letzten 6 Monate
- ○ Weiß nicht

6 **Wie oft verwenden Sie diese Produkte?**	Gar nicht	Einmal in der Woche oder weniger	Mindestens zwei Mal pro Woche	Mindestens einmal am Tag	Mindestens zweimal am Tag
Elektrische Zahnbürste	○	○	○	○	○
Handzahnbürste	○	○	○	○	○
Fluoridgel (z. B. Elmex Gelée)	○	○	○	○	○
Zahnseide	○	○	○	○	○
Interdentalbürsten	○	○	○	○	○

7 **Wie lange putzen Sie Ihre Zähne in der Regel?**

- ○ Etwa eine halbe Minute oder weniger
- ○ Etwa eine Minute
- ○ Etwa zwei Minuten
- ○ Etwa drei Minuten
- ○ Etwa vier Minuten oder mehr

Wissen über Mundgesundheit

*In diesem Abschnitt werden Sie gebeten, Fragen zu verschiedenen Themen zu beantworten, die sich auf Ihr **Wissen über Mundgesundheit** beziehen (z.B. Ernährung, Mund-/Zahnanatomie, Hygiene, etc.).*

---

8 **Wie gut kennen Sie sich mit Themen aus, die die Mundgesundheit betreffen?**

○ Ausgezeichnet ○ Sehr gut ○ Gut ○ Mäßig ○ Schlecht

---

9 **Sind Säuren (z.B. im Orangensaft) schädlich für die Mundgesundheit?**

○ Nein
○ Ja, denn sie verursachen Parodontitis
○ Ja, denn sie hinterlassen Zahnbelag-Rückstände
○ Ja, denn sie verursachen Erosionen des Zahnschmelzes
○ Weiß nicht

---

10 **Bitte kreuzen Sie in der folgenden Liste die drei Lebensmittel an, die für die Mundgesundheit am schädlichsten sind.**

○ Apfelsaft ○ Karotten ○ Schokolade
○ Schwarzer Tee (ohne Zucker) ○ Weichkäse ○ Kuchen

---

11 **Wann ist der beste Zeitpunkt, sich nach dem Verzehr von Obst die Zähne zu putzen?**

○ Es ist überhaupt nicht notwendig, sich danach die Zähne zu putzen
○ Unmittelbar danach
○ 30 Minuten danach
○ Frühestens zwei Stunden danach
○ Weiß nicht

---

12 **Wie oft wechseln Sie die Zahnbürste oder den Bürstenkopf?**

○ Ca. jeden Monat
○ Ca. alle 3 Monate
○ Ca. alle 6 Monate
○ Ca. einmal im Jahr
○ Seltener als einmal im Jahr

---

13 **Was wird empfohlen, direkt nach dem Zähneputzen zu tun?**

○ Schaum ausspucken und Mund ausspülen
○ Nur den Schaum ausspucken, aber *nicht* ausspülen
○ Weiß nicht

---

14 **Was ist der Hauptzweck der Verwendung von fluoridhaltigen Produkten?**

○ Fluorid schützt vor Entzündungen
○ Fluorid tötet Bakterien ab
○ Fluorid verhindert Zahnbelag
○ Fluorid stärkt den Zahnschmelz
○ Fluorid macht Zähne weißer
○ Weiß nicht

---

15 **Was ist der Hauptzweck von Zahnversiegelungen (ZV)?**

○ Durch ZV werden durch Karies entstandene Löcher gefüllt.
○ ZV stärken den Zahnschmelz, um Karies zu verhindern.
○ ZV reagieren mit der Zahnpasta, dadurch wird das Zähneputzen effektiver.
○ ZV verhindern, dass sich Bakterien in den feinen Zahnrillen festsetzen.
○ Weiß nicht

16 **Welche Behandlung wird bei der Entzündung eines Zahnnervs in der Regel zuerst durchgeführt?**

○ Füllung der Wurzelkanäle
○ Versiegelung der Zahnfissuren
○ Entfernung des Zahnnervs/Gewebes
○ Entfernen der Wurzelspitzen
○ Weiß nicht

17 **Was ist eine Gingivitis? Sie ist eine….**

○ Gaumenentzündung
○ Mandelentzündung
○ Zahnfleischentzündung
○ Speicheldrüsenentzündung
○ Weiß nicht

18 **Forschungsergebnisse zeigen einen Zusammenhang zwischen Mundgesundheit und anderen Erkrankungen. Welche anderen Erkrankungen spielen dabei eine Rolle?**

○ Hauterkrankungen
○ Herzinfarkt
○ Psychische Erkrankungen
○ Muskelschwund
○ Weiß nicht

19 **Wie heißt der Teil des Zahnes, auf den der Pfeil zeigt?**

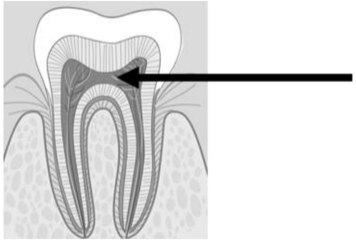

○ Zahnmark (Pulpa)
○ Zahnbein (Dentin)
○ Zahnzement
○ Zahnschmelz
○ Zahnfach (Alveole)
○ Weiß nicht

20 **Was ist ein „Bonus-Heft“?**

○ Ein Formular, das die Krankengeschichte dokumentiert.
○ Eine Informationsbroschüre, die die zahnärztlichen Leistungen der Krankenkasse beschreibt.
○ Ein Formular, das dokumentiert, wie oft ich zu einer Kontrolluntersuchung gegangen bin.
○ Ein Dokument, das alle zahnärztlichen Leistungen einer Zahnzusatzversicherung beschreibt.
○ Weiß nicht

21 **Haben Sie ein “Bonus-Heft”?**

○ Ja ○ Nein ○ Weiß nicht

Wissen über Zahnbehandlung

22 **Welche der folgenden zahnmedizinischen Leistungen werden von der gesetzlichen Krankenkasse in der Regel bezahlt?** (Mehrfachantwort möglich)

- ☐ Betäubungsspritze, wenn wegen Karies gebohrt werden muss
- ☐ Implantat
- ☐ Kontrolluntersuchungen (bis zu 2 mal pro Jahr)
- ☐ Zahnfleischbehandlungen
- ☐ Zahnsteinentfernung (1 mal pro Jahr)
- ☐ Weiß nicht

23 **Der Zahnarzt ist verpflichtet vor jeder Versorgung mit Zahnersatz (Kronen, Brücken, Prothesen) einen Heil- und Kostenplan zu erstellen.**

- ○ Richtig
- ○ Falsch
- ○ Weiß nicht

24 **Im Falle einer schmerzhaften Behandlung haben Sie das Recht auf eine Betäubung.**

- ○ Richtig
- ○ Falsch
- ○ Weiß nicht

25 **Wer ist dafür verantwortlich, dass ich Kontrolluntersuchungen beim Zahnarzt wahrnehme?**

- ○ Meine Krankenkasse
- ○ Ich selbst
- ○ Mein Hausarzt
- ○ Mein Zahnarzt
- ○ Weiß nicht

Fragen zu Ihrer Person

26 **Sie sind? …** ○ männlich ○ weiblich

27 **In welchem Jahr sind Sie geboren?** Geburtsjahr: ✎ |___|___|___|___|

28 **Welchen Familienstand haben Sie?**

○ verheiratet ○ ledig ○ geschieden ○ verwitwet ○ in Partnerschaft

29 **Haben Sie Kinder?**

○ nein
○ ja, und zwar: ✎ |_____| (Anzahl Kinder)

30 **Welche Staatsbürgerschaft/en haben Sie?** (Mehrfachantwort möglich)

☐ Deutsch
☐ andere, und zwar: ✎

31 **Seit wann haben Sie die deutsche Staatsbürgerschaft?**

○ seit Geburt
Seit dem Jahr … : ✎ |___|___|___|___|

32 **Sind Sie in Deutschland geboren?** ○ ja (weiter mit Frage 33) ○ nein

a **In welchem Land sind Sie geboren?** Name des Landes: ✎

b **Seit wann leben Sie in Deutschland?** Jahr Ihrer Einreise: ✎ |___|___|___|___|

33	**Wo wurden Ihre Eltern geboren?**		
a	Mutter	○ Deutschland<br>○ anderes Land: ________________	○ weiß nicht
b	Vater	○ Deutschland<br>○ anderes Land: ________________	○ weiß nicht
34	**Wie viele Jahre sind Sie insgesamt zur Schule gegangen?**	Anzahl der Jahre: |___|___|	

35 **Welchen höchsten Schulabschluss oder Hochschulabschluss haben Sie?**

○ noch in der Schule	○ Realschulabschluss/Mittlere Reife
○ ohne Schulabschluss abgegangen	○ Fachhochschulreife/Abitur
○ Haupt-/Volksschulabschluss	○ Fachhochschulabschluss/Hochschulabschluss
○ einen anderen Schulabschluss, und zwar:	

36 **Sind Sie zurzeit erwerbstätig?** ○ ja ○ nein

Falls ja:

○ Vollzeit angestellt (mind. 35 Std./Woche)	○ Teilzeit angestellt (weniger als 35 Std./Woche)
○ selbstständig / freiberuflich	○ angestellt *und* freiberuflich

Falls nein:

○ als arbeitssuchend gemeldet	○ Ausbildung/Umschulung/Studium
○ Erwerbs-/Berufsunfähigkeitsrente	○ Hausfrau/Hausmann
○ Alters-/Frührente	○ *keine* Arbeitserlaubnis
○ anderer Grund, und zwar:	

37 **Wie sind Sie krankenversichert?**

○ gar nicht versichert	○ gesetzlich
○ privat	○ beihilfeberechtigt
○ sonstige:	

38 **Wie viele Personen <u>einschließlich Sie selbst</u> leben in Ihrem Haushalt?** |___|___|

39 **Wie hoch ist das monatliche Haushaltsnettoeinkommen, d.h. das Nettoeinkommen, das Sie (alle zusammen) nach Abzug der Steuern und Sozialabgaben haben?** *Gemeint sind alle Einkünfte aus Arbeit, Rente, Sozialhilfe, Vermietung, Kindergeld, Pflegeversicherung und anderen Quellen.*

○ unter 1.000 €	○ 3.000 € bis unter 3.500 €
○ 1.000 € bis unter 1.500 €	○ 3.500 € bis unter 4.000 €
○ 1.500 € bis unter 2.000 €	○ 4.000 € bis unter 5.000 €
○ 2.000 € bis unter 2.500 €	○ über 5.000 €
○ 2.500 € bis unter 3.000 €	○ Weiß nicht

40 **Welche Sprache wird in Ihrem Haushalt überwiegend gesprochen?**

41 **Wie gut können Sie die deutsche Sprache …?**

	sehr gut	gut	es geht	eher schlecht	gar nicht
… lesen	○	○	○	○	○
… sprechen	○	○	○	○	○

42 **Falls Sie nicht so gut Deutsch sprechen: Haben Sie schon einmal einen Übersetzer zum Arzt/Zahnarzt mitgenommen?**

○ Ja, einen professionellen Dolmetscher
○ Ja, ein Familienmitglied/Freund
○ Nein, ich hätte einen Übersetzer gebraucht, aber habe keinen gefunden
○ Nein, mein Deutsch ist grade gut genug

43 **Wie würden Sie Ihren gegenwärtigen Gesundheitszustand beschreiben?**

sehr gut	gut	zufriedenstellend	weniger gut	schlecht
○	○	○	○	○

44 **Hat ein Arzt bei Ihnen jemals eine oder mehrere der folgenden Krankheiten festgestellt?**

☐ nein	☐ Diabetes	☐ Asthma	☐ Herzkrankheit
☐ Krebserkrankung	☐ Schlaganfall	☐ Migräne	☐ Bluthochdruck
☐ depressive Erkrankung	☐ Gelenkerkrankung (Arthrose, Rheuma)	☐ chronische Rückenbeschwerden	☐ Schlafstörung

☐ sonstige Erkrankung(en):

45 **Wie zufrieden sind Sie gegenwärtig, alles in allem, mit Ihrem Leben?**

Antworten Sie bitte anhand der folgenden Skala, wobei "0" ganz und gar unzufrieden und "10" ganz und gar zufrieden bedeutet.

Ganz und gar <u>un</u>zufrieden ⓪ ① ② ③ ④ ⑤ ⑥ ⑦ ⑧ ⑨ ⑩ Ganz und gar zufrieden

Falls Ihre Eltern und Sie selbst in Deutschland geboren und aufgewachsen sind, überspringen Sie bitte die folgenden 4 Fragen. Falls Sie oder Ihre Mutter und/oder Vater aus einem anderen Land stammen, beantworten Sie bitte auch diese letzten Fragen.

46 **Wie stark fühlen sie sich in die Gesellschaft in Deutschland integriert?**

Gar nicht ⓪ ① ② ③ ④ ⑤ ⑥ ⑦ ⑧ ⑨ ⑩ Sehr stark

47 **Wie stark fühlen Sie sich mit Deutschland verbunden?**

Gar nicht ⓪ ① ② ③ ④ ⑤ ⑥ ⑦ ⑧ ⑨ ⑩ Sehr stark

48 **Wie stark fühlen Sie sich mit Ihrem bzw. dem Herkunftsland Ihrer Eltern verbunden?**

Gar nicht ⓪ ① ② ③ ④ ⑤ ⑥ ⑦ ⑧ ⑨ ⑩ Sehr stark

49 **Wie häufig haben Sie persönlich innerhalb der letzten beiden Jahre die Erfahrung gemacht, hier in Deutschland aufgrund ihrer Herkunft benachteiligt worden zu sein?**

○ häufig ○ selten ○ nie

Vielen Dank für die Beantwortung des Fragebogens! 😉

Bitte kreuzen Sie abschließend an: Haben Sie den Fragenbogen…?

○ selbstständig oder ○ mit Hilfe ausgefüllt? Datum: ________________
